# Identifying labour market pathways after a 30-day-long sickness absence –a three-year sequence analysis study in Finland

**DOI:** 10.1186/s12889-023-15895-2

**Published:** 2023-06-07

**Authors:** Riku Perhoniemi, Jenni Blomgren, Mikko Laaksonen

**Affiliations:** 1 The Social Insurance Institution of Finland, +358504072270 Nordenskiöldinkatu 12, Helsinki, 00250 Finland; 2 Finnish Centre for Pensions, Helsinki, Finland

**Keywords:** Long-term sickness absence, Work disability, Disability pension, Rehabilitation, Labour market state, Sequence analysis, Longitudinal study, Register study, Clustering

## Abstract

**Background:**

Return-to-work (RTW) process often includes many phases. Still, multi-state analyses that follow relevant labour market states after a long-term sickness absence (LTSA), and include a comprehensive set of covariates, are scarce. The goal of this study was to follow employment, unemployment, sickness absence, rehabilitation, and disability pension spells using sequence analysis among all-cause LTSA absentees.

**Methods:**

Register data covered full-time and partial sickness allowance, rehabilitation, employment, unemployment benefits, and permanent and temporary disability pension (DP), retrieved for a 30% representative random sample of Finnish 18–59 years old persons with a LTSA in 2016 (N = 25,194). LTSA was defined as a ≥ 30-day-long full-time sickness absence spell. Eight mutually exclusive states were constructed for each person and for 36 months after the LTSA. Sequence analysis and clustering were used to identify groups with different labour market pathways. In addition, demographic, socioeconomic, and disability-related covariates of these clusters were examined using multinomial regressions.

**Results:**

We identified five clusters with emphases on the different states: (1) rapid RTW cluster (62% of the sample); (2) rapid unemployment cluster (9%); (3) DP after a prolonged sickness absence cluster (11%); (4) immediate or late rehabilitation cluster (6%); (5) other states cluster (6%). Persons with a rapid RTW (cluster 1) had a more advantaged background than other clusters, such as a higher frequency of employment and less chronic diseases before LTSA. Cluster 2 associated especially with pre-LTSA unemployment and lower pre-LTSA earnings. Cluster 3 was associated especially with having a chronic illness before LTSA. Those in cluster 4 were on average younger and had a higher educational level than others. Especially clusters 3 and 4 were associated with a LTSA based on mental disorders.

**Conclusions:**

Among long-term sickness absentees, clear groups can be identified with both differing labour market pathways after LTSA and differing backgrounds. Lower socioeconomic background, pre-LTSA chronic diseases and LTSA caused by mental disorders increase the likelihood for pathways dominated by long-term unemployment, disability pensioning and rehabilitation rather than rapid RTW. LTSA based on a mental disorder can especially increase the likelihood for entering rehabilitation or disability pension.

**Supplementary Information:**

The online version contains supplementary material available at 10.1186/s12889-023-15895-2.

## Background

With high financial and human costs, occupational disability is a major challenge for today’s societies. Numerous studies have shown that tackling disability as early as possible is essential. The longer the sickness absence period continues, the lower is the probability for returning to sustained full-time work, and the higher the chance for a disability pension transition [1–3].

Once a long-term sickness absence (LTSA) occurs, personal characteristics such as younger age, higher education and higher socioeconomic position are known to enhance the chances for return-to-work (RTW) and decrease the risk for prolonged or permanent disability [4–6]. However, it is important to unravel not only risk factors for disability, but also different pathways from sickness absence back to work or to other labour market states. The RTW is often not a straightforward process, but instead includes many phases of alternating labour market positions. Transitions between paid sick leave, treatment and rehabilitation periods, employment, and unemployment may alternate after the initial sickness absence period [7–9].

Sequence analysis has proven to be a valuable method in capturing the different phases and transitions involved in the RTW process [4, 5, 9–12]. However, sequence analyses or other multi-state analyses have often concentrated on distinct diagnosis groups [4, 10, 13] or have had a limited standpoint to the states studied with focus on either labour market attachment [10, 11] or disability benefits [4]. Sequence analyses with all-cause LTSA absentees, that include labour market states central to RTW (employment, unemployment, sickness absence, rehabilitation, and disability pension) on the one hand, but also include a comprehensive set of background characteristics on the other, are scarce. Only Madsen [5] and Pedersen et al. [9] have conducted sequencing and clustering of a diverse set of relevant states for individuals on all-cause LTSA, identifying groups with emphases on RTW or dependency on temporary or permanent social benefits. Madsen’s study also showed the association of older age, lower education and lower occupational class with labour market pathways characterized by unemployment, reliance on support or prolonged disability.

Knowledge on the frequency and timing of rehabilitation is a central issue associated with early support and RTW. However, the timing of rehabilitation has often been lacking in multi-state models identifying post-LTSA labour market transitions. Madsen’s [5] and Pedersen’s et al. [9] studies have identified groups with successful, late, and prolonged rehabilitation and temporary support. Finnish retrospective register studies have shown that the use of vocational rehabilitation can be insufficient before entering the disability pension process [14, 15], indicating a need to further understand the timing of rehabilitation once LTSA occurs.

In addition, the pathways after LTSA can depend on a wide range of personal demographic and socioeconomic characteristics, and baseline labour market position [4, 5, 9, 11, 12]. While the medical condition behind LTSA obviously can play a central role in the process, the role of LTSA diagnosis for future paths has not been widely accounted for in the multi-state models. Thus information on the diagnosis for LTSA and the pre-existing chronic diseases must also be integrated in the study.

The aim of this study was to follow the alteration between employment, unemployment, further sickness absence spells, rehabilitation, and disability pension after LTSA, using sequence analysis and objective register data. We aimed to identify clusters based on the individual sequences, and to examine covariates of these clusters using a wide range of background characteristics.

## Methods

### Study population and follow-up

All persons who started a full-time sickness absence spell lasting at least 30 days during 2016 were first retrieved from a fully representative random sample of the Finnish population, including 30% of all working-age persons. LTSA was thus defined as a ≥ 30-day-long full-time sickness absence spell.

The study sample was then restricted to 18–59 years old persons with no sickness absence days during 12 months prior to that spell (N = 25,194). The age limits were set so that all the subjects would be of adult age and would not reach the lowest limit of old-age pension in Finland (63 years) during the follow-up. Subjects were followed for 36 months (three years) from the first day exceeding 30 LTSA days.

Data on full-time and partial sickness allowance spells, demographics and the existence of chronic or severe diseases were retrieved from registers of the Social Insurance Institution of Finland (Kela). Data on socioeconomic status was obtained from Statistics Finland. Employment and unemployment benefit spells, and annual earned incomes were retrieved from registers of the Finnish Centre for Pensions. Rehabilitation spells and benefits were retrieved from Kela and the Finnish Centre for Pensions. Rehabilitation included vocational rehabilitation (e.g. work try-outs, training), medical rehabilitation (e.g. physiotherapy, multidisciplinary rehabilitation), discretionary rehabilitation (e.g. adaptation training, courses) and rehabilitative psychotherapy. All data on benefit and employment spells included start and end dates. Data on temporary and permanent disability pensions was derived from registers of Kela and the Finnish Center for Pensions, including the start dates of DP.

### Disability and rehabilitation benefits in Finland

Sickness absence was measured through compensated sickness allowance days. Kela can pay sickness allowance to non-retired persons aged 16–67 as compensation for loss of income due to sickness or impairment. The allowance can be paid when the sickness absence exceeds 10 working days, covered by the employer. A physician’s sickness certificate is needed for the allowance. Based on a certain diagnosis, the allowance can generally be paid up to twelve months during two years’ time. Partial sickness allowance can be granted if work ability is reduced but the beneficiary is able to continue working part-time.

A disability pension may mainly be granted after the statutory maximum period of full-time sickness allowance. A temporary disability pension can be granted to compensate earnings loss during rehabilitation or treatment. Also the rehabilitation benefit is meant for securing income during vocational or medical rehabilitation that is already realizing or secured.

Register data on sickness allowance spells included the start and end dates and diagnoses of the spells. Persons with full-time sickness absence spells at least 30 days long were studied, as longer sickness absence both signals the need for care or rehabilitation and are a more significant risk for permanent disability [1–3, 16]. Although there is no universal definition of LTSA, the definition of 30 sickness absence days has been used in multiple studies [17–20].

### Definition of the states

Eight mutually exclusive states were constructed for each person and for each of the 36 months of the follow-up. The possible states in each month were permanent disability pension, rehabilitation, temporary disability pension, full sickness allowance, partial sickness allowance, unemployment or employment. If none these sources of income could be found for a 1-month unit, the state of that month was recorded as other/unknown. These other states included those who either died during the follow-up (1.9%, N = 476) or exited the Finnish population (0.3%, N = 82).

In the case of overlap, the state mentioned earlier in the above list dominated over the states listed later. An exception was made in the case where there was both unemployment and employment ‒ and no states “above” them ‒ during a 1-month unit. In that case the state was defined based on which of the two states had more registered days.

### Covariates

The nine covariates were mostly measured in the start of 2016. Age was classified into four groups (see Table 1). Marital status was categorized as married, unmarried, and divorced, separated or widowed. Socioeconomic status was measured in terms of educational level and occupational class. Educational level was categorized into upper tertiary, lower tertiary, secondary and primary education. Occupational class distinguished between upper and lower non-manual employees, manual workers, entrepreneurs, and others following the classification of Statistics Finland [21]. The occupational class “other” included the long-term unemployed, students and persons without a statistical classification. Labour market status at the start of the LTSA was defined as employed, unemployed or other. Income from earnings in 2015 was divided into quartiles. Entitlement to reimbursements for medicine expenses was used as a proxy measure for chronic or severe diseases in the start of 2016 [22]. These entitlements are ensured through National Health Insurance and guarantee the recipients’ access to medicines needed for the treatment of certain long-term diseases at a reasonable cost. The study population was also classified based on the diagnosis of their first LTSA spell in 2016 according to the ICD-10 classification [23]. Diagnosis groups were mental disorders (‘mental LTSA’), musculoskeletal diseases (‘musculoskeletal LTSA’), and ‘other diagnosis LTSA’.

### Statistical methods

Sequence analysis was used to study the temporal succession of states and to summarize the intertemporal variation between individuals [24]. Sequences were defined as 36-month strings of the states. Successive months with the same state formed *episodes*. To illustrate the proportional and individual changes in the eight states over time, status proportion plots and sequence index plots were created. To further examine sequences and changes, the frequencies of states, and total durations and the average number of episodes for each of the eight states were drawn from the created sequences. Furthermore, the average number of transitions *in total* and the average number of *different* states in the sequences were examined. The Stata SQ-Ados [24] and SADI packages [25] were used for the analyses and graphs.

Individual sequences were grouped into clusters [26] based on inter-sequence distances. Optimal matching analysis (OMA) was applied to calculate the inter-sequence distances between individual sequences [27], with substitution costs set at double the size of indel cost [24]. Cluster analysis was conducted with Ward’s linkage [24]. Point Biserial Correlation (PBC), Average Silhouette width (ASW), Hubert’s C coefficient (HC), Calinski-Harabasz pseudo-F’s index (CH) were used as cluster cutoff criteria [28, 29]. Finally, multinomial regression analysis, with odds ratios and their 95% confidence intervals, was used to analyze covariates associated with belonging to each cluster.

### Ethical considerations

In Finland, an ethical review statement is not required for studies based solely on administrative register data [30]. We followed good scientific practice, data protection guidelines and ethical standards in collecting and analysing the data and in reporting the results. Permissions to use pseudonymised register data were obtained from the original data holders.

**Table 1:** The covariates in the study sample (N = 25,194)

	N	%
Sex		
Male	11,089	44.0
Female	14,105	56.0
Age group		
18–30	5,455	21.7
31–40	5,701	22.6
41–50	6,550	26.0
51–59	7,488	29.7
Marital status		
Married	11,182	44.4
Unmarried	10,094	40.1
Divorced / separated / widowed	3,918	15.6
Educational level		
Upper tertiary	2,956	8.2
Lower tertiary	5,472	21.7
Secondary	13,440	53.5
Primary	4,226	16.8
Occupational class		
Upper non-manual employee	3,041	12.1
Lower non-manual employee	7,877	31.3
Manual worker	6,647	26.4
Entrepreneur	1,809	7.2
Other	5,820	23.1
Labour market status at the start of LTSA		
Employed	19,689	78.2
Unemployed	3,473	13.8
Other	2,032	8.1
Earnings income 2015		
1st quartile	6,299	25.0
2nd quartile	6,298	25.0
3rd quartile	6,299	25.0
4th quartile	6,298	25.0
Chronic diseases		
No	16,770	66.6
Yes	8,424	33.4
LTSA diagnosis group		
Mental LTSA	3,910	15.5
Musculoskeletal LTSA	4,314	17.1
Other diagnosis LTSA	16,970	67.4
Total	**25,194**	**100.0**

## Results

### Proportions and duration of states and transitions between states

**Fig. 1:**
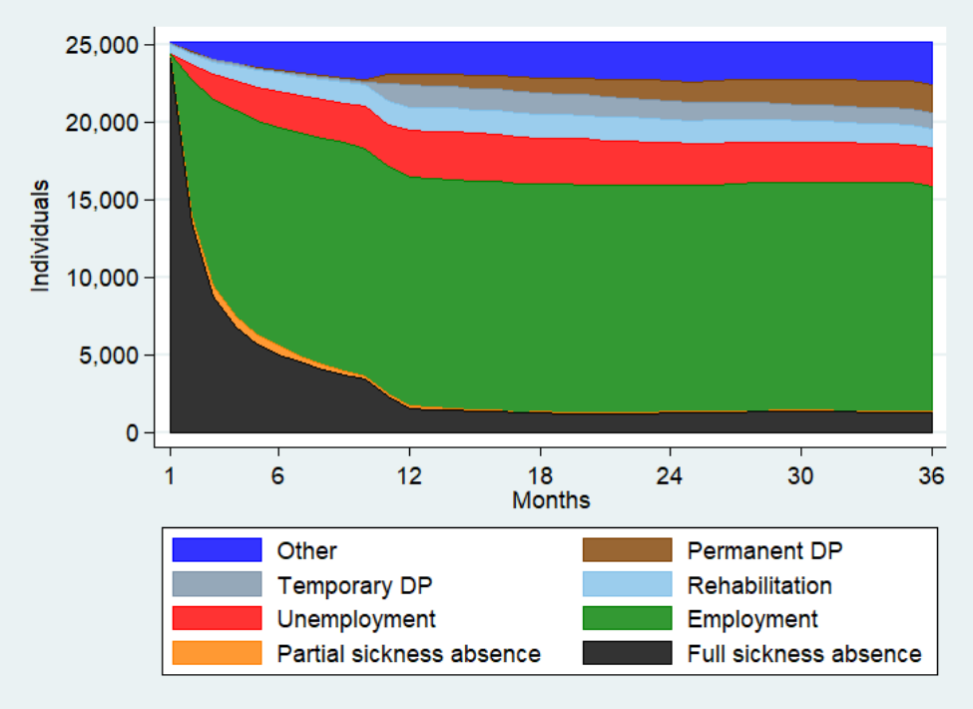
Status proportion plot visualizing the relative proportion of the states

Figure 1 visualizes the proportion of individuals in each state in the 36 follow-up months. The proportion of persons on full sickness allowance decreased radically from the first follow-up months, and stayed stable from the 12th follow-up month. Employment was by far the most frequent state after the first few follow-up months. After 12 months, 59% were employed and the proportion remained very stable for the rest of the follow-up. Also the proportion of persons unemployed increased until the 12th follow-up month and stayed stable there on. After 12 months, frequencies of permanent and temporary DP started to increase, as in this point many persons reach the statutory maximum period of full sickness absence. Rehabilitation periods occurred from the first month on and the proportion remained stable to the end of the follow-up. Partial sickness allowance was not frequent and occurred mostly during the first 12 months. The proportion of those in some other unknown state was rather stable over time.

In general, the average number of transitions between states during the 36-month follow-up was 4.0 (standard deviation [SD] 1.9). While only one transition was most typical in all sequences (25.4% of the study sample), 45.9% had four or more transitions during the follow-up. The average number of *different* states in a sequence was 2.8 (SD 0.9).

**Table 2:** Frequency, average duration and average number of episodes of the states

	At least one episode	Average total duration (months)	Average number of episodes	Status in the final (36th ) month
		Mean (Sd)	Mean (Sd)	
Permanent DP	7.3%	1.32 (5.28)	0.07 (0.26)	7.3%
Temporary DP	10.0%	1.30 (4.73)	0.13 (0.45)	4.0%
Rehabilitation	18.9%	1.93 (5.77)	0.36 (0.88)	4.9%
Unemployment	27.0%	3.62 (7.88)	0.52 (1.06)	9.7%
Employment	82.5%	20.0 (13.34)	1.69 (1.36)	57.9%
Partial sickness absence	9.4%	0.26 (0.94)	0.11 (0.35)	0.3%
Full sickness absence	97.8%	4.52 (3.92)	1.58 (0.93)	4.9%
Other/unknown	31.7%	3.05 (6.62)	0.51 (0.93)	11.0%

Table 2 provides more details on the frequencies, proportions and total durations of the states and episodes. In total, 7% of the long-term sickness absentees in 2016 transferred to full DP during the follow-up. One out of ten (10%) had a temporary DP, and 19% had a rehabilitation period at some point. Partial sickness allowance was used by 9%. Most (83%) were employed at some time point, whereas unemployment was experienced by 27%. A third of the study sample (32%) had at least one month with some other, unknown state during the follow-up.

Examining the average total durations of each state, employment covered on average the most, 20.0 months of the follow-up. Full sickness absence (mean 4.5 months), unemployment (mean 3.6 months) and other states (mean 3.1) were the next most frequent states, with rehabilitation (mean 1.9 months), temporary DP (mean 1.3 months), and permanent DP (mean 1.3 months) coming next. Respectively, the average number of episodes was also highest for employment (mean 1.7) and full sickness absence (mean 1.6).

### Clusters of individual state trajectories

Out of four cluster-stopping indexes, three supported a five-cluster solution (see supplementary table S1, highest value on PBC and ASW, and lowest value on HC). In addition, the five-cluster solution distinguished between clear cluster identities with different emphases of the examined states, and was thus chosen.

**Fig. 2:**
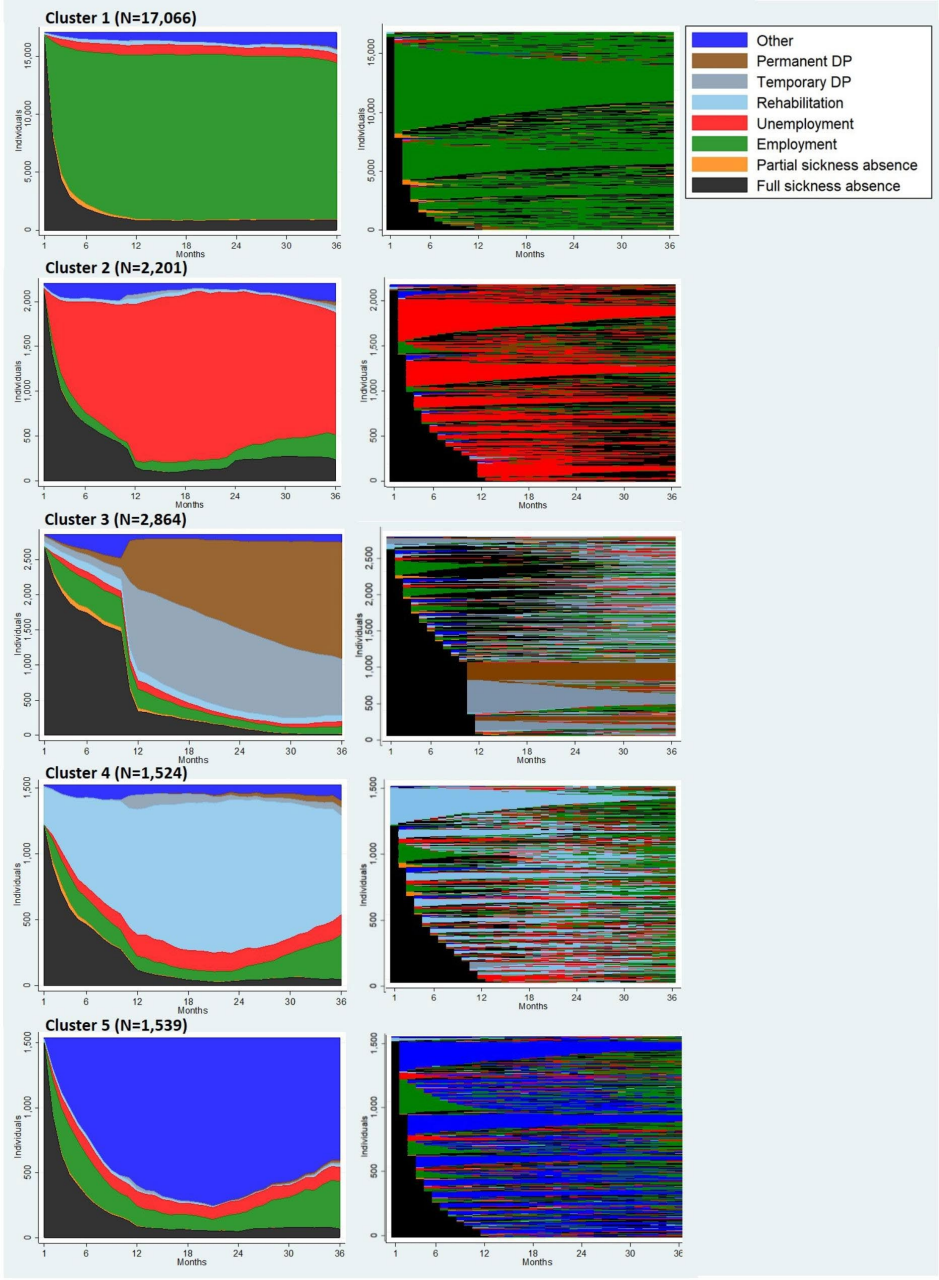
Status proportion plots visualizing the relative proportion of each states for each cluster (left) and sequence index plots visualizing individual sequences in each cluster (right) over the follow-up

Both status proportion plots and sequence index plots were produced to illustrate the clusters (Fig. 2). The sequence index plots visualize how fragmented many applicants’ labour market pathways are. To further characterize the five clusters, aggregated sequence characteristics (supplementary table S2) and ten most frequent sequence patterns (supplementary table S3) were described for each cluster.

Cluster 1 (67.7%), the largest one, was dominated by **rapid RTW** both in long total duration of employment (on average 28.1 months) and higher average number of employment episodes (2.1). The cluster also depicted RTW with little rehabilitation or temporary DP (table S2). For a large portion of persons in this cluster, a rather short full-time sickness absence was followed by an unbroken or a long-lasting employment period lasting to the end of the follow-up (Fig. 2). For the rest, employment after the initial 30-day LTSA was cut by other states, mostly by new full sickness absence spells. In fact, while this cluster had the lowest average months of full sickness absence in total, they had on average the highest amount of separate full sickness absence spells and often alternated between employment and full sickness absence (table S3).

Cluster 2 (8.7%) included persons with emphasis on **rapid unemployment**. Many faced prolonged unemployment that lasted on average 24.6 months of the whole 36 month follow-up time (S2). A significant proportion of persons in this cluster had a long unemployment period right after the initial 30-day LTSA, and 15% were only unemployed alter LTSA for the whole follow-up (table S3). However, the majority alternated between unemployment and other states during the follow-up (Fig. 2), and there was a high average number of transitions (4.3). Those other states were often full sickness absence, employment or some other, unknown state (table S3).

Cluster 3 (11.4%) consisted mostly of individuals transferring to either permanent or temporary **disability pension after a prolonged sickness absence** spell (Fig. 2). The average overall duration of full sickness absence after the initial 30-day LTSA in this cluster was higher than in other clusters, 7.8 months (table S2). Respectively, 27.9% were on temporary and 58.2% were on permanent DP at the end of the follow-up (not presented in tables). Roughly half transitioned to temporary or permanent DP after an unbroken chain of full sickness absence spells (Fig. 2). For many though, sickness absence was followed by other states before the DP transition, oftentimes employment or an unknown state (Fig. 2, table S3). Overall, the average number of different states during the follow-up was high (3.4). Average months in rehabilitation was 1.4, which is more than in clusters 1, 2 and 5. Employment during the follow-up was less frequent than in the other clusters (table S2). For 19.4%, there was a transition from temporary to permanent DP (not presented in tables).

Cluster 4 (6.1%), a rather small one, included persons with **immediate or late rehabilitation**. The average overall duration of rehabilitation during the follow-up was 21.6 months (table S2). Although for many, the rehabilitation started early in the follow-up, the proportion of persons in rehabilitation was most frequent around the 24th follow-up month (Fig. 2). 49.0% were in rehabilitation and 22.3% were employed at the end of the follow-up (not presented in tables). Persons in this cluster had on average 2.2 separate rehabilitation spells. Compared to other clusters, these persons had on average the largest number of transitions (5.3), and number of different states (3.5) during the follow-up.

Finally, cluster 5 (6.1%), included persons with a lot of months in **other states** during the follow-up, i.e. unknown states that were not captured by the variables available in this data set. Of the measured states, employment, unemployment and new full sickness absence spells altered most often with the unknown states (Fig. 2; table S2).

Besides forming its own cluster, the states not captured by our study design were present in other clusters. They were usually placed between full sickness absence and the next states, especially before unemployment (cluster 2), DP (cluster 3) or rehabilitation (cluster 4), as seen in Fig. 2.

### Associations between the covariates and cluster membership

Finally, we used multinomial logistic regression analysis to examine how the demographic, socioeconomic and disability-related covariates were associated with the cluster membership (Table 3). The rapid RTW cluster (cluster 1) was chosen as the base outcome in the model, since it was the largest, reflected the societally preferred route after sickness absence, and was the most homogeneous in its contents. In the analysis, clusters 2 to 5 are not directly compared to each other but always to cluster 1. Supplementary table S4 also shows covariate frequencies in each cluster.

There were some similarities between clusters in how the covariates associated with cluster memberships. In relation to the base outcome cluster 1, all four clusters 2 to 5 were associated with not being employed at the start of the initial LTSA, lower pre-LTSA earnings and having a chronic illness before the LTSA and a LTSA based on a mental disorder. Clusters 2, 3, and 5 were associated with having only primary level education, while clusters 2 and 3 were associated with older age.

In addition, *rapid unemployment* cluster 2 membership was associated especially with in the occupational class “other”, pre-LTSA unemployment, and lower pre-LTSA earnings. There was also a mild, although statistically significant association with male sex. *Disability pension after a prolonged sickness absence* cluster 3 was associated especially with having a chronic illness before LTSA and having a LTSA based on a mental disorder. *Immediate or late rehabilitation* cluster 4 had the most unique profile: In relation to rapid RTW cluster 1, the likelihood for belonging to cluster 4 were increased by female sex, younger age, and higher education level. Like with cluster 3, a LTSA based on a mental disorder raised especially the likelihood for cluster 4 membership. Finally, in addition to factors presented above, the likelihood for belonging to *other states* cluster 5 were raised by age 18 to 30 and labour market status “other” at the start of the initial LTSA.

**Table 3:** Associations between covariates and cluster memberships. Multinomial logistic regression model, cluster 1 as reference category. All variables adjusted for (N = 25,194). OR = odds ratio; CI = confidence interval

	Cluster 2: rapid unemployment		Cluster 3: DP after a prolonged sickness absence		Cluster 4: immediate or late rehabilitation		Cluster 5: other states	
	**OR**	**95% CI**	**OR**	**95% CI**	**OR**	**95% CI**	**OR**	**95% CI**
Sex								
Male	1,00		1,00		1,00		1,00	
Female	0,86*	0,77‒0,97	**0,90**	0,82‒0,99	1,35***	1,19‒1,53	1,06	0,94‒1,19
Age group								
18–30	1,00		1,00		1,00		1,00	
31–40	1,92***	1,62‒2,28	1,32**	1,11‒1,57	0,95	0,82‒1,13	0,61***	0,52‒0,72
41–50	2,49***	2,09‒2,97	2,13***	1,80‒2,52	0,81	0,68‒0,97	0,44***	0,37‒0,54
51–59	3,32***	2,77‒3,97	5,70***	4,84‒6,72	0,56***	0,46‒0,68	0,68***	0,59‒0,82
Marital status								
Married	1,00		1,00		1,00		1,00	
Unmarried	1,14	0,99‒1,30	0,95	0,85‒1,07	1,00	0,87‒1,15	1,05	0,91‒1,22
Divorced / separated / widowed	1,40***	1,20‒1,62	1,20**	1,06‒1,35	1,13	0,95‒1,34	1,26*	1,05‒1,50
Educational level								
Upper tertiary	1,00		1,00		1,00		1,00	
Lower tertiary	0,87	0,64‒1,18	0,90	0,72‒1,13	0,71**	0,57‒0,88	0,88	0,68‒1,15
Secondary	1,12	0,84‒1,50	1,16	0,93‒1,45	0,56***	0,45‒0,70	0,92	0,70‒1,20
Primary	1,70**	1,26‒2,31	1,42**	1,12‒1,79	0,39***	0,30‒0,52	1,35*	1,01‒1,80
Occupational class								
Upper non-manual employee	1,00		1,00		1,00		1,00	
Lower non-manual employee	1,04	0,75‒1,44	1,03	0,84‒1,26	0,71**	0,57‒0,88	0,64***	0,50‒0,82
Manual worker	1,32	0,95‒1,82	1,12	0,91‒1,38	0,85	0,67‒1,08	0,66**	0,51‒0,85
Entrepreneur	0,38	0,25‒0,59	0,79	0,62‒1,01	0,61**	0,45‒0,84	1,01	0,76‒1,34
Other	2,45***	1,78‒3,37	1,58***	1,26‒1,98	1,11	0,87‒1,43	0,96	0,75‒1,25
Labour market status at the start of LTSA								
Employed	1,00		1,00		1,00		1,00	
Unemployed	6,17***	5,24‒7,27	3,36***	2,87‒3,94	2,48***	2,03‒3,04	2,12***	1,74‒2,60
Other	3,52***	2,89‒4,29	3,46***	2,88‒4,16	3,90***	3,20‒4,77	5,08***	4,22‒6,11
Earnings income 2015								
1st quartile	1,00		1,00		1,00		1,00	
2nd quartile	0,36***	0,31‒0,42	0,64***	0,55‒0,74	0,63***	0,53‒0,75	0,54***	0,45‒0,64
3rd quartile	0,14***	0,11‒0,18	0,39***	0,33‒0,46	0,52***	0,42‒0,64	0,29***	0,24‒0,36
4th quartile	0,10***	0,08‒0,13	0,31***	0,26‒0,37	0,42***	0,33‒0,52	0,35***	0,28‒0,44
Chronic diseases								
No	1,00		1,00		1,00		1,00	
Yes	1,29***	1,15‒1,44	3,06***	2,79‒3,35	1,27***	1,12‒1,43	1,30***	1,15‒1,46
LTSA diagnosis group								
Mental LTSA	1,00		1,00		1,00		1,00	
Musculoskeletal LTSA	0,56***	0,47‒0,67	0,30***	0,26‒0,35	0,27***	0,23‒0,33	0,65***	0,53‒0,80
Other diagnosis LTSA	0,51***	0,44‒0,58	0,32***	0,28‒0,36	0,27***	0,24‒0,30	0,78***	0,67‒0,90

* statistically significant, p < .05; ** statistically significant, p < .01; *** statistically significant, p < .001

## Discussion

This study examined the alternation between sickness absence, employment, unemployment, rehabilitation and disability pensions following a long-term sickness absence (LTSA) spell at least 30 days long. By using sequence analysis, the primary goal was to identify groups with different emphases on these states, different transitions between states and unique covariates.

### Distinct groups based on individual sequences

Based on sequence analysis, we found five clusters with clear identities and emphases on the labour market states examined. As in previous studies [5, 9], most LTSA absentees here returned quite rapidly to employment after the 30-day LTSA spell and were mostly attached to work during the three-year follow-up. Our analysis identified this *rapid RTW* cluster as the largest in size, covering 62% of all subjects. Notably, this group mostly returned directly to work, with few records of other states or rehabilitation periods in between. Interestingly, while the rapid RTW group had the lowest average number of sickness absence months in total after the initial 30-day LTSA, they had the highest amount of separate full sickness absence spells during the follow-up. This shows that for many the fast RTW can still include new sickness absence spells before a more stable RTW. The rapid RTW group had a better socioeconomic background, i.e. a higher employment frequency, and a lower frequency of chronic diseases before LTSA than members of other clusters. Such an advantaged background naturally aids in restoring the work ability and returning to work. In addition, in Finland those employed usually have access to free-of-charge Occupational Health Services, specialized in work ability issues [31], aiding the RTW.

In contrast to the rapid RTW group, cluster 2 identified those with a quite rapid transition to unemployment after LTSA (9% of the subjects). This group had relatively scattered paths, with the second most transitions between different states, typically between unemployment and new full sickness absence spells or employment. The alteration between states and difficulty of work attachment is not surprising as unemployment or lower socioeconomic status intertwine with disability in many ways [32, 33], making full restoration of work ability hard. Long-term unemployment is strongly associated with lower health in general and can have adverse effects on work ability as well. Being in this group was indeed associated especially with pre-LTSA unemployment, a manual worker status, and lower pre-LTSA earnings.

The third cluster identified persons transitioning to permanent or temporary disability pension (11%). They were characterized especially by older age, having a chronic illness before the initial LTSA, but also by a lower socioeconomic status as shown also in previous studies [5, 16, 34, 35]. Many had used the statutory maximum length of full-time sickness absence, a precondition and a major risk factor for permanent DP transfer [36]. Our results add understanding of the process to DP, showing the frequency of other states between sickness absence and DP. While roughly half of those in this cluster transferred to DP directly from a prolonged full sickness absence [1–3] ‒ probably reflecting a severe medical condition that makes work ability very hard to restore ‒ many transferred to a permanent DP after several transitions and other states, reflecting the general complexity of the disability retirement process (e.g. [37]). For those with rehabilitation and employment spells before DP, these transitions reflect attempts to restore work ability. However, although more common than in the rapid employment and unemployment clusters, rehabilitation before DP was not very frequent. This supports earlier findings of the underutilization of rehabilitation before permanent DP [14, 15]. One in five transitioned from temporary to permanent DP during the follow-up supporting previous studies’ observation that temporary DP rather rarely leads to employment but instead often serves as a gateway to permanent pension [38–40].

The fourth cluster identified persons with immediate or late rehabilitation, whether medical or occupational (6%). Rehabilitation spells covered on average two years of the three-year follow-up. The recurrence of rehabilitation was frequent and there was a high number of transitions between different states, for example between employment and rehabilitation. It is understandable that if work disability is severe enough to require rehabilitation, the RTW process can be incoherent. Especially vocational rehabilitation spells can be long, and recurrent rehabilitation periods common [8, 41]. For many in our study, the rehabilitation started early in the follow-up. Persons in this cluster were on average young and they had a high educational level. These observation are linked, as socioeconomically privileged groups are overrepresented among the recipients of early rehabilitation [42], whereas there may be a risk for a late rehabilitation among persons with a lower socioeconomic position [5, 43]. Early intervention or rehabilitation in general have a positive effect on RTW [44]. However, in addition to early rehabilitation, many might have gone through late or delayed rehabilitation. This was indicated by the fact that almost half of this group were still in rehabilitation at the end of the follow-up. However, our analysis did not distinguish between early or successful rehabilitation and delayed rehabilitation. Nor did the results identify an independent group returning to work through rehabilitation. Females were slightly over-presented in this cluster, supporting previous Finnish studies concerning participation in rehabilitation [42, 45].

Finally, there was a fifth group (6%) whose paths after the initial 30-day LTSA were mostly unknown, i.e. dominated by states or sources of income not captured by our study. This group may include persons living on other social security benefits that our data did not capture, such as family benefits or students’ benefits. Other possibilities are living on other household members’ income, on their own savings, or on capital income. Interestingly, when examining the other clusters with more distinct identities, the unknown other state was usually placed between full sickness absence and the next state. This can reflect the difficulty to stabilize the labour market position and secure income during or after work disability. As it also may indicate a break in income, future studies could shed more light on this observation.

### Diagnostic background and early chronic diseases associated with the clusters

Sickness absentees’ background factors such as higher age and lower socioeconomic on RTW processes are well known to predict future sickness absences and disability and a lower likelihood for RTW [4, 5, 9, 11, 12]. However, less is known about the role of LTSA diagnosis for future multi-state paths. In general, mental LTSA has been associated with a lower likelihood of RTW [46]. In our study, compared to those returning rapidly to employment, all other clusters were associated with LTSA based on a mental disorder. In the only previous sequence analysis study that included mental health problems as a predictor [9], mental health problems predicted prolonged sickness absence, and less fast RTW compared to other diagnoses. In other studies, sickness absence due to mental disorders has been associated with a disability pension risk [1, 2, 47]. In Finland, mental disorders are also the most common causes behind temporary DP spells, and they decrease the likelihood for employment after the temporary DP. Instead, they increase the likelihood for a prolonged temporary DP [39, 48]. In our study, especially the rehabilitation and DP groups had a LTSA based on a mental disorder. On the one hand, an increasing global awareness of work disability caused by mental problems can increase the roll-in to psychotherapy and other rehabilitation methods. On the other hand, mental disorders continuously lead to permanent disability pensions as it is difficult to cope with mental disorders at work especially in today’s demanding working life. Better access to early preventive services supporting mental health as well as task adjustment may be some of the ways that could increase the probability of rapid return to work among those with mental challenges.

Unlike in many previous studies, we were also able to control for the pre-LTSA chronic diseases. Unsurprisingly, having at least one chronic disease before the LTSA increased especially the risk of being in the cluster of persons that transferred to disability pensions. This background factor can reflect general morbidity, and in some cases a lengthy or severe condition behind the sickness absence. A higher number of diseases, and comorbidity of somatic or psychiatric conditions increase the risk for long occupational disability [49, 50]. In any case, controlling for pre-LTSA morbidity and thus possible comorbidity does strengthen our observations concerning the predictive roles of LTSA diagnosis group, as well as other background variables.

### Strengths and weaknesses

Unlike many previous multi-state studies, we were able to study multiple relevant labour market states reflecting the RTW process after long-term work disability, and to utilize register data on all states. Furthermore, unlike many sequence analyses or other multi-state analyses on long-term sickness absence, we included a comprehensive set of register-based demographic, socioeconomic, and disability-related covariates. Registers are deemed to be highly reliable and objective, with no self-report bias and no loss to follow-up. However, the time-span is a limitation in our study. Like RTW in general, rehabilitation can be a long process, and our three-year follow-up may not have been totally sufficient to show more steady benefits from the rehabilitation spells. Our rehabilitation cluster showed persons that were in rehabilitation for the most of the three-year follow-up. As Madsen’s study has shown, it may require a long follow-up setting to distinguish between early, late or successful rehabilitation groups. In addition, our proxy measure for chronic disease was not ideal. While information in the register for special entitlements for medicine expenses has been considered to be a good proxy for morbidity [22], these entitlements are most often granted for diseases of the circulatory system, diabetes or asthma, whereas in disability benefits, the emphasis is on mental disorders and musculoskeletal diseases [51]. Furthermore, our study does not concern all working age persons with lowered work ability. Persons outside work may not apply for sickness allowance if the benefit would not raise their income level, or if they are not aware of how receiving sickness allowance can affect later entitlement for i.e. disability pension [52]. Finally, as national contexts are unique in their legislation and labour markets, it is not certain to what extent our results are generalizable to other contexts. Future international studies on similar designs could strengthen the results found here.

## Conclusions

We identified distinct groups among long-term sickness absentees, with both differing labour market pathways after a 30-day LTSA and differing backgrounds. A lower socioeconomic background, pre-LTSA chronic diseases and mental disorders increase the likelihood for long-term unemployment, disability pensioning and rehabilitation spells rather than rapid RTW. LTSA based on a mental disorder can especially increase the likelihood for rehabilitation or disability pension. Future multi-state studies could build on our observations by analyzing rehabilitation processes related to successful RTW and income gaps in RTW pathways.

## Electronic supplementary material

Below is the link to the electronic supplementary material.

Supplementary Material 1

## Data Availability

The data that support the findings of this study are available from Kela, Finnish Centre for Pensions and Statistics Finland but restrictions apply to the availability of these data, which were used under license for the current study, and so are not publicly available.

## Abbreviations

RTW: Return-to-work
LTSA: Long-term sickness absence
DP: Disability pension
Kela: Social Insurance Institution of Finland
OMA: Optimal matching analysis
PBC: Point Biserial Correlation
ASW: Average Silhouette width
HC: Hubert’s C coefficient
CH: Calinski-Harabasz pseudo-F’s index
