# Supplementary material for: Identifying labour market pathways after a 30-day-long sickness absence –a three-year sequence analysis study in Finland
Source: BMC Public Health. 2023 Jun 7;23:1102. doi: 10.1186/s12889-023-15895-2 (PMC10245454; doi:10.1186/s12889-023-15895-2)
Supplement: Supplementary file 1 — Supplementary Material 1 [file 12889_2023_15895_MOESM1_ESM.docx]

**Supplementary table S1. Point Biserial Correlation (PBC), Average Silhouette width (ASW), Hubert's C coefficient (HC), Calinski-Harabasz pseudo-F’s index (CH) cutoff criteria values.**

| \| **Number of clusters** \| \| --- \| | **PBC** | **ASW** | **HC** | **CH** |
| --- | --- | --- | --- | --- | --- |
| 2 | .716 | .473 | .080 | 8356.4 |
| 3 | .777 | .483 | .056 | 5817.8 |
| 4 | .812 | .494 | .040 | 4899.0 |
| 5 | .835 | .517 | .029 | 4449.0 |
| 6 | .754 | .430 | .054 | 4656.3 |
| 7 | .766 | .448 | .045 | 4491.6 |
| 8 | .771 | .439 | .034 | 4051.4 |

**Supplementary table S2. Aggregated sequences characteristics of five clusters.**

|  | Cluster 1: rapid RTW | Cluster 2: rapid unemployment | Cluster 3: DP after a prolonged sickness absence | Cluster 4: immediate or late rehabilitation | Cluster 5: other states |
| --- | --- | --- | --- | --- | --- |
|  | Mean (Sd) | Mean (Sd) | Mean (Sd) | Mean (Sd) | Mean (Sd) |
| **Average total duration (months)** |  |  |  |  |  |
| Permanent DP | .01 (.27) | .05 (.40) | 11.24 (11.44) | .36 (2.17) | .10 (1.09) |
| Temporary DP | .05 (.52) | .24 (1.20) | 10.32 (9.87) | .97 (2.55) | .16 (.94) |
| Rehabilitation | .59 (2.14) | .55 (1.55) | 1.38 (2.89) | 21.57 (8.09) | .38 (1.57) |
| Unemployment | 1.55 (4.19) | 24.63 (6.74) | .92 (2.83) | 2.91 (5.40) | 2.31 (4.30) |
| Employment | 28.07 (7.09) | 2.28 (3.50) | 2.52 (4.70) | 3.43 (4.25) | 4.73 (5.18) |
| Partial sickness absence | .31 (1.01) | .02 (.27) | .31 (1.06) | .18 (.76) | .07 (.49) |
| Full sickness absence | 3.72 (3.27) | 6.28 (5.39) | 7.84 (3.76) | 4.79 (4.10) | 4.50 (4.00) |
| Other/unknown | 1.70 (3.85) | 1.94 (3.43) | 1.46 (3.41) | 1.77 (3.87) | 23.76 (6.49) |
| **Average number of episodes** |  |  |  |  |  |
| Permanent DP | .00 (.06) | .02 (.14) | .58 (.49) | .03 (.18) | .01 (.10) |
| Temporary DP | .02 (.13) | .06 (.25) | 0.84 (.86) | .30 (.74) | .05 (.26) |
| Rehabilitation | .19 (.64) | .31 (.81) | .51 (.97) | 2.16 (.1.20) | .14 (.52) |
| Unemployment | .37 (.95) | 2.05 (1.20) | .18 (.52) | .72 (1.22) | .47 (.84) |
| Employment | 2.08 (1.25) | .81 (1.21) | .55 (.95) | 1.16 (1.30) | .1.21 (1.31) |
| Partial sickness absence | .13 (.37) | .01 (.09) | .11 (.38) | .09 (.31) | .03 (.18) |
| Full sickness absence | 1.65 (.96) | 1.58 (.84) | 1.40 (.82) | 1.26 (.83) | 1.41 (.76) |
| Other/unknown | .42 (.89) | .53 (.76) | 32. (.63) | .54 (.92) | 1.83 (1.07) |
| **Average number of transitions** | 3.86 | 4.31 | 3.49 | 5.27 | 4.15 |
| **Average number of different states in sequence** | 2.69 (.78) | 3.20 (.82) | 3.41 (1.02) | 3.46 (.87) | 2.55 (.71) |

**Supplementary table S3. Ten most frequent sequence patterns for clusters (order of episodes regardless of length). 1= Permanent DP; 2 = Temporary DP; 3= Rehabilitation 4 = Unemployment; 5 = Employment; 6 = Partial sickness allowance; 7 = Full sickness allowance; 8 =Other/unknown.**

| Cluster 1: rapid RTW | | | Cluster 2: rapid unemployment | | | Cluster 3: DP after a prolonged sickness absence | | |
| --- | --- | --- | --- | --- | --- | --- | --- | --- |
| **Sequence order** | **N** | **%** | **Sequence order** | **N** | **%** | **Sequence order** | **N** | **%** |
| 7 5 | 5055 | 29,6 | 7 4 | 330 | 15,0 | 7 1 | 367 | 12,8 |
| 7 5 7 5 | 1923 | 11,3 | 7 4 7 4 | 111 | 5,0 | 7 2 | 216 | 7,5 |
| 7 5 7 5 7 5 | 696 | 4,1 | 7 8 4 | 84 | 3,8 | 7 2 1 | 194 | 6,8 |
| 7 5 8 5 | 510 | 3,0 | 7 4 5 4 | 78 | 3,5 | 7 5 1 | 88 | 3,1 |
| 7 6 5 | 448 | 2,6 | 7 4 8 4 | 58 | 2,6 | 7 8 1 | 83 | 2,9 |
| 7 5 8 | 309 | 1,8 | 7 4 5 | 54 | 2,5 | 7 8 2 | 64 | 2,2 |
| 7 5 4 5 | 223 | 1,3 | 7 4 5 4 5 4 | 39 | 1,8 | 7 8 2 1 | 61 | 2,1 |
| 7 5 7 | 192 | 1,1 | 7 4 8 | 38 | 1,7 | 7 5 2 1 | 37 | 1,3 |
| 7 5 7 5 7 5 7 5 | 192 | 1,1 | 7 4 7 | 38 | 1,7 | 2 1 | 35 | 1,2 |
| 7 5 8 5 8 5 | 158 | 0,9 | 7 5 4 | 35 | 1,6 | 7 4 1 | 35 | 1,2 |
| Subtotal for ten most frequent sequences | 9,706 | 56,9 | Subtotal for ten most frequent sequences | 865 | 39,3 | Subtotal for ten most frequent sequences | 1,180 | 41,2 |
| Cluster 4: immediate or late rehabilitation | | | Cluster 5: other states | | |  | | |
| **Sequence order** | **N** | **%** | **Sequence order** | **N** | **%** |  |  |  |
| 7 3 | 59 | 3,9 | 7 8 | 267 | 17,3 |  |  |  |
| 3 5 | 25 | 1,6 | 7 5 8 | 114 | 7,4 |  |  |  |
| 7 5 3 | 23 | 1,5 | 7 5 8 5 | 52 | 3,4 |  |  |  |
| 7 4 3 | 21 | 1,4 | 7 4 8 | 44 | 2,9 |  |  |  |
| 7 3 5 | 20 | 1,3 | 7 8 5 8 | 32 | 2,1 |  |  |  |
| 7 5 3 5 3 | 17 | 1,1 | 7 5 8 5 8 5 | 31 | 2,0 |  |  |  |
| 7 3 5 3 | 16 | 1,0 | 7 8 5 | 28 | 1,8 |  |  |  |
| 7 2 3 | 16 | 1,0 | 7 5 8 5 8 | 27 | 1,8 |  |  |  |
| 7 8 3 | 15 | 1,0 | 7 8 4 | 25 | 1,6 |  |  |  |
| 3 5 3 5 | 13 | 0,9 | 7 8 4 8 | 25 | 1,6 |  | 8=Other/unknown; 1= Permanent DP; 3= Rehabilitation; 2 = Temporary DP; 6= Partial sickness allowance; 4= Unemployment; 5= Employment; 7= LTSA |  |
| Subtotal for ten most frequent sequences | 225 | 14,8 | Subtotal for ten most frequent sequences | 645 | 41,9 |  |  |  |

**Supplementary table S4. The covariate frequencies by clusters.**

|  | Cluster 1: rapid RTW | Cluster 2: rapid unemployment | Cluster 3: DP after a prolonged sickness absence | Cluster 4: immediate or late rehabilitation | Cluster 5: other states |
| --- | --- | --- | --- | --- | --- |
|  | % | % | % | % | % |
| Sex |  |  |  |  |  |
| Male | 43.7 | 49.7 | 47.2 | 33.4 | 43.8 |
| Female | 56.3 | 50.3 | 52.8 | 66.6 | 56.2 |
| Age group |  |  |  |  |  |
| 18-30 | 19.9 | 20.1 | 13.2 | 34.8 | 45.7 |
| 31-40 | 24.5 | 20.8 | 11.9 | 28.0 | 18.7 |
| 41-50 | 27.9 | 27.8 | 21.0 | 23.2 | 14.7 |
| 51-59 | 27.7 | 31.3 | 53.9 | 14.0 | 20.9 |
| Marital status |  |  |  |  |  |
| Married | 47.9 | 30.9 | 44.4 | 37.6 | 31.8 |
| Unmarried | 38.0 | 47.2 | 33.5 | 49.4 | 55.4 |
| Divorced / separated / widowed | 14.1 | 21.9 | 22.1 | 13.0 | 12.8 |
| Educational level |  |  |  |  |  |
| Upper tertiary | 9.0 | 3.5 | 5.6 | 12.3 | 6.6 |
| Lower tertiary | 24.4 | 11.2 | 16.7 | 23.6 | 15.2 |
| Secondary | 53.5 | 52.4 | 55.8 | 51.5 | 50.3 |
| Primary | 13.2 | 32.9 | 22.0 | 12.7 | 27.9 |
| Occupational class |  |  |  |  |  |
| Upper non-manual employee | 14.0 | 2.8 | 7.5 | 14.7 | 10.2 |
| Lower non-manual employee | 36.9 | 9.9 | 23.0 | 27.4 | 18.8 |
| Manual worker | 30.1 | 13.8 | 22.8 | 17.6 | 18.5 |
| Entrepreneur | 7.9 | 2.0 | 7.2 | 4.6 | 9.0 |
| Other | 11.1 | 71.6 | 39.6 | 35.7 | 43.5 |
| Labour market status at the start of LTSA |  |  |  |  |  |
| Employed | 91.8 | 26.4 | 58.2 | 62.5 | 53.9 |
| Unemployed | 5.1 | 58.3 | 29.1 | 16.6 | 15.8 |
| Other | 3.2 | 15.4 | 12.8 | 20.9 | 30.3 |
| Earnings income 2015 |  |  |  |  |  |
| 1st quartile | 12.4 | 73.8 | 40.7 | 39.7 | 51.5 |
| 2nd quartile | 26.2 | 16.3 | 25.8 | 24.1 | 23.5 |
| 3rd quartile | 30.4 | 5.8 | 18.2 | 19.2 | 11.2 |
| 4th quartile | 31.0 | 4.2 | 15.2 | 17.0 | 13.8 |
| Chronic diseases |  |  |  |  |  |
| No | 71.3 | 62.2 | 39.5 | 69.0 | 68.0 |
| Yes | 28.7 | 37.8 | 60.5 | 31.0 | 32.0 |
| LTSA diagnosis group |  |  |  |  |  |
| Mental LTSA | 10.2 | 23.8 | 25.0 | 39.2 | 21.7 |
| Musculoskeletal LTSA | 18.3 | 17.7 | 15.2 | 11.5 | 12.5 |
| Other diagnosis LTSA | 71.5 | 58.5 | 59.8 | 49.3 | 65.8 |
| Total | **100.0** | **100.0** | **100.0** | **100.0** | **100.0** |
